# The Role of Plant Cell Wall Proteins in Response to Salt Stress

**DOI:** 10.1155/2014/764089

**Published:** 2014-01-19

**Authors:** Lyuben Zagorchev, Plamena Kamenova, Mariela Odjakova

**Affiliations:** Department of Biochemistry, Faculty of Biology, Sofia University “St. Kliment Ohridski”, 8 Dragan Tsankov Boulevard, 1164 Sofia, Bulgaria

## Abstract

Contemporary agriculture is facing new challenges with the increasing population and demand for food on Earth and the decrease in crop productivity due to abiotic stresses such as water deficit, high salinity, and extreme fluctuations of temperatures. The knowledge of plant stress responses, though widely extended in recent years, is still unable to provide efficient strategies for improvement of agriculture. The focus of study has been shifted to the plant cell wall as a dynamic and crucial component of the plant cell that could immediately respond to changes in the environment. The investigation of plant cell wall proteins, especially in commercially important monocot crops revealed the high involvement of this compartment in plants stress responses, but there is still much more to be comprehended. The aim of this review is to summarize the available data on this issue and to point out the future areas of interest that should be studied in detail.

## 1. Introduction

Abiotic stress comprises a major issue for contemporary agriculture in terms of low crop yield and increasing areas not suitable for planting [1]. Among the abiotic stress factors high soil salinity and the lack of fresh water supplies are among the greatest obstacles for a high productive agriculture. Above 20% of the agricultural lands in the world are affected by high salinity and this percentage is expected to be further increased [2]. Another concern is the global climate change that leads to more and more extreme fluctuation of the environmental conditions in agricultural areas [3].

**Figure 1:**
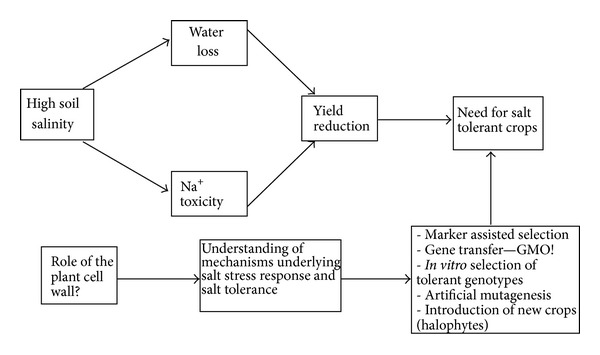
Schematic representation of the negative effects of high soil salinity on crop yield and the strategies to overcome it. The role of the plant cell wall in salt stress response and tolerance is depicted as one of the least known aspects and extensive studies in this area are needed in order to understand the mechanisms of salt tolerance and apply this knowledge to the strategies for salt tolerant crops development.

Probably, the high salt concentrations in a number of agricultural lands appear to be the major restriction for successful crop breeding, and therefore salt stress response and tolerance in plants tend to receive the greatest attention [4, 5]. Another reason is the multitrait essence of salt stress response in plants and the multiple common mechanisms with other stresses. The adverse effect of salts operates at several levels (Figure 1). The first and most obvious effect is the reduction in the osmotic potential of the soil solution thus causing reduction in plant available water, effect very similar in a biochemical aspect to the one caused by water deficit. Additionally the salt ions and especially Na^+^ are directly toxic to the plant cell. Their flux into the cell via unspecific cationic channels leads to a severe inhibition of enzymatic activities and therefore to a general breakdown of metabolic pathways [5]. High salinity could also lead to nutritional imbalance [6].

Simultaneously, there is an increasing demand for inexpensive food supply as the Earth population is growing at an extremely high rate, from 6.1 billion in mid-2001 to 9.3 billion expected by 2050 (http://www.unfpa.org/swp/2001/). Recent calculations suggest that by 2025 the food production should be increased by 35% and by 57% up to 2050. Unfortunately these rates could appear to be underestimated. As the potential for establishing new cultivation areas is exhausted, therefore it is needed to achieve higher yield from the existing ones [7].

## 2. Strategies for Improving Salt Stress Tolerance of Crops

As conventional breeding strategies could not face the on growing problem [8], it is in front of plant science on physiological and molecular level to give the answers. Various approaches (Figure 1) for screening of cultivars to select stress tolerant genotypes of the traditional crops were reviewed by Munns and James [9] and more recently by Ashraf et al. [10] and stated several contradictions between researchers. Marker assisted breeding is often discussed as the future of agriculture. It relies on quantitative trait locus (QTL) to be targeted in marker-assisted selection (MAS) [10, 11]. Despite the recent advance in that approach, the introgression of multiple QTLs into elite varieties is an expensive and laborious process. Moreover, the reported results in the case of stress-related QTLs are still limited [12]. Recent improvement of the technology extend the MAS to genomic selection (GS) [13] that is believed to overcome some of the limitations of MAS and to predict complex-traits value of a particular cultivar.

Another alternative is the production of highly resistant crops using the methods of plant biotechnology and gene transfer. It possesses a great potential especially with the possibility for multigenic transfer thus enabling the introduction of whole metabolic pathways [14]. However, genetically-modified crops generally encounter mistrust from both governments and society. A less contradictory alternative is using fast *in vitro* selection and micropropagation of stress tolerant genotypes that is giving some promising results in a variety of crops [15, 16]. Salt tolerance may be also induced by artificial mutagenesis of *in vitro* cultures [17], usually by gamma rays.

Whatever will be the future strategy, it is of crucial significance to understand how plants respond to environmental stresses (Figure 1) as organisms and on cell and molecular level? In recent years, a lot was done in that aspect and there are numerous studies exploring these processes [5, 18, 19]. However, there is still much more to be elucidated. Most of the investigations are conducted on model plants, with the dicotyledonous *Arabidopsis thaliana* being the plant of choice. The extrapolation of this knowledge on important crop plants could lead to some success but more and more studies should be directed to economically important species.

Monocots comprise the major part of the daily food supply with maize, wheat, and rice being the most important. Reduction in growth due to abiotic stress and especially high salinity was reported for many of them [20]. The understanding of their metabolic response to environmental stresses could seriously improve agriculture. As there are a lot of differences from species to species even if they are closely related and either from cultivar to cultivar [21], this task is further complicated. From cereals rice is reported to be the most sensitive to salt stress and barley the most resistant [22]. These differences in stress tolerance should be annotated to substantial differences in the proteome. Moreover, commercial crops seem to exhibit much less effective mechanisms to tolerate stress factors compared to their wild relatives [23]. That is the reason wild relatives are also regarded as a “gene pool” for crops tolerance improvement [24]. Another potentially fruitful area of investigations is orientated towards halophytes and the mechanisms underlying their extreme tolerance to high salt concentrations [25]. Besides being an obvious genetic resource for stress tolerant features, halophytes are also regarded as the possible “crops of the future” [26].

## 3. Emerging Role for the Plant Cell Wall

The role of the plant cell wall in plant life emerged with the compartment to compartment proteomics. It appeared that it is not only a physical barrier between the plant cell and the environment but also a very flexible and responsive part of the cell, functionally involved in growth and differentiation, signaling and response to pathogenic attack, and different stresses [27]. It is composed of about 95% carbohydrates with cellulose, pectin, and hemicelluloses being the major ones. Also there is a significant amount of proteins both structural and functional of which role in plant cell stress response is arising in recent years. Very few thorough studies on the cell wall proteome of several monocots as maize [28] and rice [29] were conducted in recent years, but still most of the data is based on *Arabidopsis* [30]. However, a significant part of these proteins remain with elusive function. About 11% of the cell wall proteins from *Arabidopsis thaliana* are referred to as with “unknown function” in a summary of a number of proteomics studies [31]. The percentage for less studied plants including cereals could be expected to be much higher. Even when certain protein is known and established to be upregulated by abiotic stresses, the exact function remains unknown.

Studying plant cell wall proteins appeared to be a tough task. The obtaining of a pure fraction is usually complicated due to the complexity of the cell wall structure, the manner of binding of the proteins, and the probable contamination from intracellular proteins. Furthermore, the great variation of posttranslational modifications and especially the abundant glycosylation makes the plant cell wall protein fraction extremely complex and difficult to investigate [31]. Another issue is the comparatively low percentage of cell wall proteins from the whole cell proteome that impede the isolation of large amount of material, needed for a thorough investigation especially of low abundant proteins.

## 4. Proteins of the Cell Wall

The establishment of an exhaustive classification of CWP is not an easier task than the choice of an appropriate experimental methodology. The first attempts for comprehensive reviews in the mid- and late nineties were focused mainly on hydroxyproline and glycine rich proteins such as extensins, arabinogalactan proteins, and lectins [32, 33]. Further in the investigation of CWP a number of proteins with enzymatic or signaling activity emerged. The most recent classification proposed subdivision of CWP into nine different classes [30] including proteins, related to the lipid and carbohydrate metabolism, proteins with oxido-reductive and proteolytic enzymatic activities, proteins, involved in signaling and molecular interactions, structural proteins, miscellaneous proteins, and protein with unknown functions. Classification of CWP is, however, further rendered difficult due to the multiple functions of some of them. For example AGPs are currently regarded as signaling proteoglycans, but also as molecules that link the cell wall with the plasma membrane and the cytoskeleton, putatively structural function [34]. In another class of structural proteins, the extensins are essential for the primary cell wall architecture [35] but belong to the same class as AGPs, the hydroxyproline rich proteins, and several authors reported chimeric AGPs that share similar structural components as extensins [36, 37].

For the purpose of the present review we will focus on particular CWP with functions in stress response and tolerance that is already established or strongly suggested disregarding the affiliation to particular class.

### 4.1. Stress Receptors on the Cell Wall

**Figure 2:**
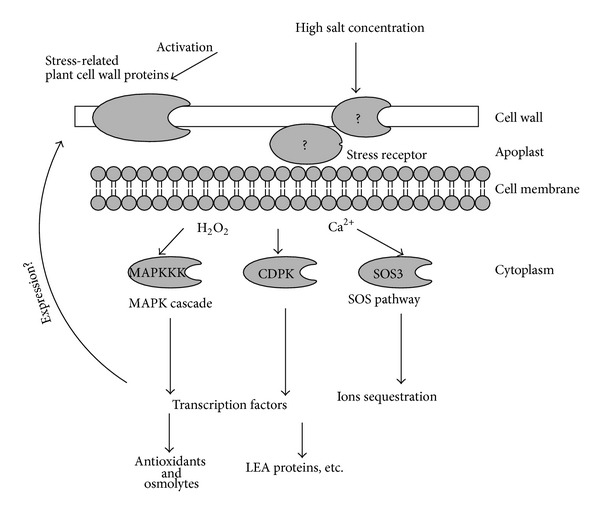
Perception and signaling during salt stress response. The major pathways inside the cell (MAPK, CDPK, and SOS) are well known. Most of them are Ca^2+^-dependent, but oxidative signaling involving H_2_O_2_ is also an important mechanism that is currently better understood. Still the data on possible involvement of cell wall receptors like cell wall associated kinases are scarce and need to be elucidated.

The signal cascades during abiotic stress response in plants were extensively studied and most of the major players were identified (Figure 2). These include mitogen-activated protein (MAP), salt overly sensitive (SOS) kinases, phospholipases, and transcription factors (e.g., heat shock factor (HSF) and the C-repeat-binding factor/dehydration-responsive element binding protein (CBF/DREB), and ABA-responsive element binding factor/ABA-responsive element (ABF/ABRE) families), all reviewed by Vinocur and Altman [38]. While several comprehensive reviews on signaling during abiotic stress response are available [39, 40], still little is known about the function of the plant cell wall in stress perception and downward signaling cascades (Figure 2). Being the first barrier between the plant cell and its surrounding environment, it is probable to expect the presence of different stress reception mechanisms localized in the cell wall. The situation may be far more complicated than expected as there is strong evidence for different kinases to be activated in response to different levels of the same stress factor [41].

The family of wall-associated kinases was widely investigated as potential candidate for a cell wall “sensor” [42]. Twenty-six WAK and WAK-like genes were identified in Arabidopsis [43]. In comparison a total of 125 OsWAKs genes were identified in rice, a substantial and one of few expansions of gene family in the monocot plant [44]. Generally WAKs are tightly bound to the pectic network of the cell wall, protrude the membrane, and link it to the cytoplasm where a Ser/Thr kinase domain is responsible for further signaling [42]. The role of WAKs in rice plant defense against pathogens is well established [45], but involvement in abiotic stress response is also proposed. In addition to WAKs a wide variety of cell wall kinases like Pro-rich extensin-like receptor kinases (PERKs), lectin receptor kinases (LecRKs), and leucine-rich repeats receptor-like kinases (LLR) RLKs were recently identified and reviewed [46]. At least twelve different receptor-like kinases from different organisms were proved to be abiotic stress responsive [40]. As the above cited reports suggest that the main stress perception-to-signaling event seems to occur at the cell wall-plasma membrane interface where AGPs and some other proteins may also interact besides receptor-like protein kinases [47].

An interesting aspect of the cell wall stress perception and signaling properties is the production of hydrogen peroxide and downward redox signaling. Reactive oxygen species and the redox potential of the intracellular environment were recently established as important and variable signaling component with extremely vital function in plant growth, development, and stress response [48]. Although chloroplasts and mitochondria are referred as the main players in these events, it seems that some CWP may be also involved in the initial ROS signaling. Hydrogen peroxide may be produced in the cell wall by activation of NADPH oxidases and cell wall peroxidases, generally in response to pathogens attack [49]. Voothuluru and Sharp [50] showed increased H_2_O_2_ accumulation in the apoplast of water stressed maize roots supposedly due to increased oxalate oxidase activity. Not only a role for ROS in cell wall loosening, but also a signaling role inside the cells was proposed. Furthermore H_2_O_2_ could transmit signals between cells, transported across the membranes by aquaporins that may be also of significant importance for abiotic stress response [51]. Production of hydrogen peroxide by NADPH oxidase is strongly influenced also by abiotic stress factors as high NaCl concentration and involved in determining tolerance to abiotic stress [52]. An interesting hypothesis suggests that inhibition of apoplastic peroxidase caused by Ca^2+^ depletion leads to H_2_O_2_ accumulation further triggering signaling events in response to a variety of stressors [53]. Assuming all stated above it seems that extracellular ROS accumulation in response to abiotic stressors is tightly regulated by a variety of cell wall bound and plasma membrane enzymes and is crucially important for stress tolerance.

### 4.2. The Multiple Functions of HRGPs

Nevertheless they were discovered comparatively a long time ago that HRGPs still remain an enigma for plant science. They are found in every plant and algae species and appeared to be involved in almost every substantial process in plant life including growth and development, embryogenesis, cell-to-cell contacts, and programmed cell death. They are localized mainly in the plasma membrane and particularly abundant in the plant cell wall [54, 55]. The general classification states three classes of HRGP according to the characteristic repetitive structural motifs yielding different degrees of O-glycosylation—extensins, arabinogalactan proteins (AGPs), and proline-rich proteins (PRPs). Among them AGPs are considered the most glycosylated with approximately 90% of carbohydrate moiety and only 10% of protein compound—the protein core [54].

**Figure 3:**
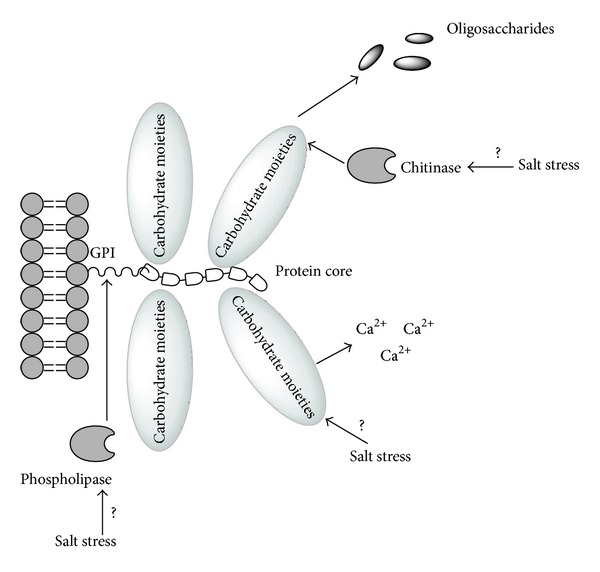
Three possible roles of the highly glycosylated arabinogalactan proteins (AGPs). (1) A specific phospholipase may cleave the glycosylphosphatidylinositol anchor (GPI) thus releasing AGPs into the cell wall and in the extracellular space. (2) AGPs may serve as storage molecules for Ca^2+^ and release them in response to different stimuli thus activating Ca-dependent signal cascades. (3) N-acetylglucosamine containing carbohydrate branches may be targets for the action of plant chitinases thus releasing oligosaccharides with signaling or osmotic adjustment functions.

The superfamily of AGPs [34, 56] consists of highly O-glycosylated and highly heterogenic proteoglycans. They could be also divided into several classes—classical AGPs, Lys-rich AGPs, AG peptides, fascilin-like AGPs (FLAs), nonclassical AGPs, and “chimeric” AGPs [57]. The apparent diversity of this class of surface proteoglycans suggests their extensive role in plant growth and development. AGPs are found at the cell surface, attached to the outer surface of the plasma membrane or bound to the cell wall. Accordingly, AGPs may contain a C-terminal glycosylphosphatidylinositol (GPI) anchor signal sequence and found attached to the plasma membrane though not being classical membrane proteins [58]. The cleavage of the GPI anchor by specific phospholipase (Figure 3) due to cellular signals and their release into the cell wall and the extracellular space seem to be of great importance in many processes [59]. These include cell development, cell death, and cell-to-cell signaling, but the specifics of their mode of action remain elusive [60]. Upregulation of the extracellular concentrations of AGPs in response to salt stress was reported in suspension *in vitro* cultures of a variety of plant species [59, 61]. Similarly increase in the concentration of HRGPs in the cell wall is also linked to tolerance to other stresses such as heavy metals [62].

Most of the studies on AGPs structure and function are investigated in *Arabidopsis* with very few identified in wheat and rice [63]. Ma and Zhao [64] identified 69 AGP-encoding genes in the rice genome. Their extensive study is the first obvious indicator for the important role of AGPs in monocots. Similarly to the previous studies they divide the supposedly expressed in rice AGPs into seven subfamilies with eNod-like AGPs and nsLTP-like AGPs included into the “chimeric” AGPs subfamily. Most of them have analogues in the *Arabidopsis* genome with only a group of nonspecific lipid transfer like AGPs absent and therefore probably existing only in monocots. In the same study most of the existing AGPs genes are proved to be expressed and a total of 15 are shown to be significantly up- or downregulated in response to abiotic stress, ABA, or GA treatments. The expression of the great variety of AGPs genes is supposed to be strictly regulated in response to different stimuli and the resulting proteoglycans should play distinct role in these processes. Still the exact molecular mechanism of action remains unknown. A suggestion that the large carbohydrate chain serves as a source for oligosaccharides that after deglycosylation increase the intracellular osmotic pressure (Figure 3) and decrease the speed of dehydration during osmotic stress was expressed [64], but even if a possible explanation, it could be still only one of the many roles of AGPs in plant stress response and probably not the main. More recently evidence for AGPs to serve as flexible storage molecules for Ca^2+^ (Figure 3) was published [65] thus suggesting another possible explanation for the stress responsive functions of AGPs as extracellular Ca^2+^ concentrations are crucial for various signaling events [40, 53]. Finally hydrolysis of the carbohydrate chains by chitinases (Figure 3) and possible signaling function of the resulting oligosaccharides were also suggested in the process of somatic embryogenesis [66]. As plant chitinases also play important role in abiotic stress response [67], it would not be unexpected that the same process occurs also in response to unfavorable environmental conditions.

Unlike AGPs, the other major classes of HRGPs, the extensins, are predominantly structural proteins that comprise scaffold for the self-assembly of the carbohydrate components of the cell wall [68]. According to several authors [69–71], extensins undergo peroxidase-mediated oxidative cross link during pathogen infection thus decreasing the permeability of the cell wall. Similar effect was also proposed for at least some AGPs classes [72]. Evidences for the same mechanism in conditions of salinity treatments were not published recently. There is, however, scarce data on salt stress responsive upregulation of extensin gene in Populus [73].

### 4.3. Cell Wall Modifying Proteins

Being the first barrier to environmental stresses the cell wall should response fast and reliable by changing its structure or composition. A number of functional proteins with enzymatic activity were found in the plant cell wall including enzymes involved in cell wall formation and reorganization, carbohydrate metabolism, and cell wall loosening. Their importance for abiotic and biotic stress adaptation was recently reviewed by Sasidharan et al. [74].

The main class of cell wall loosening CWP is that of the expansins. These relatively small molecules, around 30 kDa proteins, play a crucial role in plant growth and development. All four known groups—*α* and *β* expansins and expansin like A and B—share similar activity though quite different in amino acid sequences, and substrate specificity [75]. Their main function is associated with plant cell growth, proliferation, abscission, senescence or adaptation to stress conditions in response to variety of plant hormones [76]. Considering that the rigidness of the cell wall under unfavorable conditions is of crucial importance for stress adaptation, it is not surprising that expansins are differentially regulated in an organ specific manner and with differences from salt-sensitive to salt-tolerant cultivars in conditions of salt stress. For example, six expansin isoforms were investigated in resistant and tolerant maize cultivars and they showed differences in up- or downregulation in both cultivars [77]. The overall conclusion from the experiment is that expansins tend to keep or increase their abundance in salt-treated tolerant cultivars compared to salt-sensitive ones. Such differences may explain why salt-sensitive cultivars showed severe inhibition of growth, while salt-tolerant cultivars were able to maintain stable fresh mass accumulation even at high NaCl concentrations.

It was also proposed that differential upregulation of particular expansins isoforms is not sufficient to compensate the downregulation of others [77]. Similarly Wu et al. [78] and Sabirzhanova et al. [79] showed that fast and organ-specific (roots and leaves resp.) changes in expansin expression are osmotically induced and of crucial importance for the organism-level response to drought stress. Recent findings suggest that expansins are not only responsive elements in growth regulation under salt stress but could also confer tolerance. An example is *RhEXPA4* from rose that significantly increased salt and drought tolerance when over-expressed in Arabidopsis [80], results similar to what was established for overexpression of *TaEXPB23* in transgenic tobacco in conditions of drought stress [81]. Both results suggest that expansins maintain higher root growth and overall development of the root system (and possibly due to smaller leaves, reducing water losses) thus increasing the availability of water. In a recent experiment [82] *TaEXPB23* was expressed under stress-inducible rather than constitutive promoter and drought tolerance was also achieved but at a lower degree correlating with less changes in the phenotype.

While expansins action is directed to cellulose and xyloglucans, other CWP are responsible for changes in the pectic network of cell walls. Pectins are major components of the plant cell wall though not so highly presented in monocots, 5–10% in graminaceous compared to above 30% in dicots and nongraminaceous monocots of the wall [83]. The specific pectic epitopes occur in restricted patterns of distribution [84] and highly affect cell wall properties [85]. They can be both methyl esterified and acetyl esterified at random places along the polymer chains. A number of enzymes belonging to the esterase family like pectin methylesterases (PME; EC 3.1.1.11) and acetylesterases (AE; EC 3.1.1.6) serve as esterification and deesterification mechanism. As pectin is important for the cell wall structure and could be modified in response to different signals, pectin-modifying enzymes receive major interest in scientific studies [86].

Under stress conditions the concentration of methylated pectic epitopes tends to drop down [87] thus enabling the cross-link between pectin and Ca^2+^ which in turns lead to solidification of the wall and decreased growth [88]. The action of PME is needed for this to happen. However, PME could be also positive regulators of growth as shown for Arabidopsis hypocotyls [89]. A mutation in the promoter of PME inhibitor protein in Arabidopsis leads to increased primary root growth and biomass accumulation in conditions of salt stress [90]. Despite the recent interest on PME and PME inhibitor proteins, it seems that this class of CWP leads to controversial conclusions even when referring to the better studied importance for wound and pathogen resistance [91].

## 5. Conclusions and Future Perspectives

The role of the plant cell wall in every substantial process in plant growth and development is emerging in recent years and the involvement of the cell wall proteins as the functionally active component gains great attention. It is clear that those proteins are important in stress response and tolerance and along with the thoroughly investigated intracellular mechanisms could provide the necessary knowledge to overcome the negative impact of the environment on agriculture. Therefore, more and more studies on the changes in the structure and function of the cell wall are conducted. But does the increasing quantity of evidences leads to improved quality of our understanding on the intimate mechanisms of stress response? It seems that since now the available data is posing more questions than answers given. Nevertheless, the fast developing technology is able to provide more and more powerful tools for investigation. A complex investigation on the transcriptome, proteome, and metabolome of halophytes, relative to commercial crops, is needed for a thorough understanding of mechanisms underlying salinity tolerance. Plant cell wall proteome is inevitable player in the molecular events, following the initial response and further adaptation to stress. The perception of stress signals along with downward signaling events should be of special interest in future studies.
